# Screening and early diagnosis of chronic obstructive pulmonary disease: a population study

**DOI:** 10.1186/s12890-023-02728-6

**Published:** 2023-11-03

**Authors:** Wenhui Tang, Yan Rong, Hongmei Zhang, Wenji Lin, Wenmei Zeng, Wenhong Wu

**Affiliations:** Department of Respiratory & Critical Medicine, Shenzhen Municipal Qianhai Shekou Free Trade Zone Hospital, No. 36, Shekou Industrial seventh Road, Nanshan District, Shenzhen, 518067 China

**Keywords:** Chronic obstructive pulmonary disease, Early diagnosis, Screening

## Abstract

**Background and objective:**

Although chronic obstructive pulmonary disease (COPD) is a common disease leading to further morbidity and significant mortality, there is still limited data on screening for COPD. The purpose of this study was to establish an early chronic obstructive pulmonary disease (COPD) screening system for the community and hospitals in Nanshan District in Shenzhen City, to improve the rate of early diagnosis and treatment of patients with COPD.

**Methods:**

We identified individuals at high risk of COPD using a questionnaire survey and analyzed the relevant influencing factors in the early stages of COPD in high-risk groups.

**Results:**

We collected a total of 5,000 COPD screening questionnaires, and a total of 449 patients were diagnosed with COPD by pulmonary function examination. The prevalence of COPD in people aged 20 and above in Nanshan District of Shenzhen City was estimated to be 8.98%, with a base of 5000. The severity classification as per the Global Initiative for Chronic Obstructive Lung Disease (GOLD) criteria was as follows: GOLD I accounted for 34.74%; GOLD II accounted for 37.64%; GOLD III accounted for 16.04%; and GOLD IV accounted for 11.58%. Common features of early COPD that we identified were: (1) patients were mainly males, accounting for 68.0%; (2) COPD was common among people aged 50–59 years, comprising 31%; (3) 96.0% of patients often had severe respiratory symptoms and had frequent coughs when they did not have a cold; (4) 57.2% of patients experienced shortness of breath when walking quickly on level ground or climbing gentle slopes; (5) 72.6% of patients had a family history of bronchial asthma and COPD. Multivariate ordinal multi-classification logistic regression showed that gender, age, shortness of breath, and the use of firewood, grass, and coal stoves were all influencing factors in pulmonary function grading.

**Conclusion:**

A screening questionnaire combined with a pulmonary function test should be adopted as a COPD screening strategy to be implemented at the primary level as a public health priority in China to reduce the incidence, disability, and mortality from COPD.

## Introduction

Chronic obstructive pulmonary disease (COPD), a heterogeneous pulmonary condition characterized by chronic respiratory symptoms, is caused by persistent airflow obstruction due to airway and/or alveolar abnormalities, [1] associated with high morbidity, disability, and mortality. [2] Currently, as there is no internationally accepted definition of early COPD and no relevant information, data must be extrapolated from known patients with mild to moderate COPD. [3] A large proportion consists of patients with grade I and grade II severity as per the Global Initiative for Chronic Obstructive Lung Disease (GOLD) criteria with mild symptoms, which should be considered the early stages of COPD. [4].

Growth in COPD continues due to the rapidly increasing smoking rates in developing countries in addition to the ageing population in developed countries, prevention, early diagnosis and early treatmen of COPD is a worldwide concern. To date, data on COPD are ambiguous, and so have not resulted in the widespread implementation of similar screening for COPD in most health economies, including China [5]. In order to fill this gap, the Shenzhen Qianhai Shekou Free Trade Zone Hospital launched a public health project titled “Screening and Early Diagnosis of Chronic Obstructive Pulmonary Disease” on March 27, 2022. On November 15, 2022, Professor Chen Rongchang led the launch of the “Happy Breathing” China COPD Standardized Graded Diagnosis and Treatment Promotion Project in Shenzhen, which is based on the Nanshan District Medical Group Respiratory Disease Prevention and Treatment Regional Discipline Alliance. It established an early COPD screening system, promoted the integration of medication and prevention, and improved the rate of early diagnosis and treatment of patients with COPD by screening high-risk populations.

The purpose of this study was to report the results of an early COPD screening system for the community and hospitals in Nanshan District in Shenzhen City and to analyze the factors influencing the early stages of COPD associated with the high-risk population.

## Materials and methods

### Screening participants

We randomly selected six of the 79 social health centers in Shenzhen Nanshan District Medical Group for the study. There were regular scheduled consultations as well as unscheduled free consultations and talks. We distributed 5,000 copies of the chronic obstructive pulmonary disease screening questionnaire (COPD-SQ) to permanent residents aged 20 years and above in Nanshan District of Shenzhen City through WeChat QR code scanning and on-site form-filling. In accordance with the principle of informed consent for clinical research, we obtained written informed consent from all participants. We obtained approval from the Ethics Committee of the hospital before the implementation of the project (Ethics approval number: 2022 − 107 (Shen)-01 K).

### COPD-SQ design and identification of high-risk individuals with COPD

We developed the COPD Screening Questionnaire (Table 1) based on the criteria recommended by the *Chinese Guidelines for the Diagnosis and Treatment of COPD Disease (2021 Revised Edition)* [6] and the *Guidelines for Primary Level Diagnosis and Treatment of COPD Disease (2018).* [7].

**Table 1:** COPD screening questionnaire

Questions	Options and scoring criteria
Your age (years)	20–49(0), 50–59(3), 60–69(7), ≥ 70(10)
Your consumption of cigarettes (packs per year)	0–14(0),15–30(1), ≥ 30(2)
Your BMI (Kg/m2)	< 18.5(7), 18.5–23.9(4), 24.0-27.9(1), ≥ 28.0(0)
Do you cough often when you do not have a cold?	Yes (3), No (0)
Do you usually feel shortness of breath?	No (0), feeling shortness of breath when walking quickly on level ground or climbing gentle slopes (2), feeling shortness of breath when walking at a normal pace on level ground (3)
Do you currently use coal stoves or firewood for cooking or heating?	Yes (1), No (0)
Is there a family history of bronchial asthma, or COPD	Yes (2), No (0)

The questionnaire includes observation indicators such as age, smoking index, body mass index (BMI), respiratory symptoms (such as cough and dyspnea), family history of respiratory disease, and so on. These indicators were selected based on the criteria of high-risk groups of COPD as defined in the *Guidelines for Primary Level Diagnosis and Treatment of COPD Disease (2018)*, and those with COPD-SQ scores ≥ 16 were designated as COPD high-risk individuals.

### Diagnostic criteria for early COPD

Patients who meet the GOLD 2023 inclusion criteria with Grade I or Grade II pulmonary function (forced expiratory volume in the first second (FEV1)/forced vital capacity (FVC) < 0.7 after inhalation of bronchodilators, FEV1% pred ≥ 50%, negative result of bronchodilation test).

### Exclusion criteria (patients who meet at least one of the following criteria)

(1) patients with additional pulmonary diseases such as bronchiectasis, lung tumors, tuberculosis sequelae (destroyed lung), giant bullae, and so on; (2) patients with additional heart diseases such as dilated cardiomyopathy, unstable angina, malignant arrhythmia, and so on; (3) women who were pregnant, breastfeeding, or planning to become pregnant.

### Steps in the protocol implementation included

(1) Strengthening the training of COPD knowledge for primary-level physicians in Nanshan District of Shenzhen City, popularizing and promoting techniques for diagnosis and treatment, screening of high-risk groups for COPD disease, and training in relevant knowledge for standardized diagnosis and long-term follow-up management, thereby increasing the number of general practitioners who specialize in respiratory diseases. (2) Collecting data from 5,000 permanent residents aged 20 and above in Nanshan District of Shenzhen City using the COPD-SQ. (3) Identifying individuals at high risk of COPD using screening questionnaires and bringing them to the social health center for additional questionnaire surveys by primary doctors, with an emphasis on quality control. (4) Transferring patients to a higher-level hospital for a standard pulmonary function test, including total lung volume, residual air volume, functional residual air volume, spirometry, FEV1/FVC, diastolic testing, etc. (5) Creating medical files, offering interventions, and conducting follow-up for patients who were newly diagnosed with COPD.

### Statistical analysis

We used R (version 4.1.2) for statistical analysis. Descriptive analysis: Measurement data conforming to a normal distribution were expressed as mean ± standard deviation (X ± SD), whereas measurement data not conforming to a normal distribution were expressed as the median (interquartile range, IQR). Qualitative data were expressed as frequency (percentage). Comparison between high-risk groups: We used the t-test when the quantitative data of both groups conformed to the normal distribution; otherwise, the Mann-Whitney U test was used. We used the Chi-square test or Fisher’s exact test for qualitative data. For identifying the influencing factors of pulmonary function grading in COPD high-risk groups, we first performed univariate ordinal multi-classification logistic regression analysis on all factors to identify possible potential influencing factors for further multivariate analysis.

## Results

### Basic description of questionnaire survey results (5000 cases)

#### Basic information collected using the questionnaire

We collected a total of 5000 responses to the COPD-SQ, and the basic information is summarized as follows: ① Gender distribution: there were 3043 males (60.9%) and 1957 females (39.1%); ② Age distribution: 2700 participants (54.0%) were aged 20–49 years, 1498 participants (30.0%) were aged 50–59 years, 536 participants (10.7%) were aged 60–69 years, and 266 participants (5.3%) were aged 70 years and above; ③ Consumption of cigarettes: 3281 participants (65.6%) smoked up to 14 packs per year, 1292 participants (25.8%) smoked 15–30 packs per year, and 427 participants (8.5%) smoked ≥ 30 packs per year; ④ BMI: 583 participants (11.7%) had a BMI < 18.5 kg/m^2^, 3256 participants (65.1%) had a BMI between 18.5 and 23.9 kg/m^2^, and 1161 participants (23.2%) had a BMI between 24.0 and 27.9 kg/m^2^; ⑤ Responses to the item “Do you cough often when you do not have a cold?” were “no” in 3567 participants (71.4%), “yes” in 1428 participants (28.6%), and 5 participants did not answer the question; ⑥ For the item “Do you usually feel shortness of breath?” 428 participants (8.6%) reported that they did not have shortness of breath, 1994 participants (39.9%) felt shortness of breath when walking quickly on level ground or climbing gentle slopes, and 2578 participants (51.6%) felt shortness of breath when walking at a normal pace on level ground; ⑦ For the item “Do you currently use coal stoves or firewood for cooking or heating?” 4471 participants (89.4%) answered “no” and 528 participants (10.6%) answered “yes”, while 5 participants did not answer the question; ⑧ For the item “Do any of your parents, siblings, or children have bronchial asthma, chronic bronchitis, emphysema, or COPD?”, the answer was “no” in 2999 participants (60.0%) and “yes” in 2001 participants (40.0%). Overall score: mean (SD): 9.04 (4.54); median (Q1, Q3): 8.00 (6.00, 11.00); min–max: 0.00–28.00; normality test *P* value < 0.001. (Table 2)

**Table 2:** Basic information collected in the questionnaire

Variable	Total (N = 5000)	Variable	Total (N = 5000)
Gender		Shortness of breath	
Female	1957 (39.1%)	1	428 (8.6%)
Male	3043 (60.9%)	2	1994 (39.9%)
Missing	0	2	2578 (51.6%)
**Age**		Missing	0
1	2700 (54.0%)	**Use coal stove firewood**	
2	1498 (30.0%)	No	4471 (89.4%)
3	536 (10.7%)	No	528 (10.6%)
4	266 (5.3%)	Missing	1
Missing	0	**Family history**	
**Consumption of cigarettes**		No	2999 (60.0%)
1	3281 (65.6%)	Yes	2001 (40.0%)
2	1292 (25.8%)	Missing	0
3	427 (8.5%)	**Overall score**	
Missing	0	Mean (SD)	9.04 (4.54)
**BMI**		Median (Q1, Q3)	8.00 (6.00, 11.00)
1	583 (11.7%)	Min - Max	0.00–28.00
2	3256 (65.1%)	Missing	0
3	1161 (23.2%)	**Normality test** ***P*** **value**	< 0.001
Missing	0		
**Cough and produce phlegm without a cold**			
No	3567 (71.4%)		
Yes	1428 (28.6%)		
Missing	5		

#### COPD prevalence

Based on pulmonary function tests, a total of 449 patients were diagnosed with COPD. The prevalence of COPD in people aged 20 years and older in Nanshan District of Shenzhen City was estimated to be 8.98%, with a base of 5000.

#### Classification of severity as per GOLD criteria

There were 156 cases of GOLD I COPD, accounting for 34.74%; 169 cases of GOLD II COPD, accounting for 37.64%; 72 cases of GOLD III COPD, accounting for 16.04%; and 52 cases of GOLD IV COPD, accounting for 11.58%. Among them, there were 325 individuals with early COPD, accounting for 72.38%.

### Common features in patients with early COPD

We identified the following common features of early COPD: (1) Patients were mainly males, accounting for 68.0%; (2) Early COPD was common among people aged 50–59 years, accounting for 31%; (3) 96.0% of patients often had severe respiratory symptoms and had frequent coughs when they did not have a cold; (4) 57.2% of patients experienced shortness of breath when walking quickly on level ground or climbing gentle slopes; (5) 72.6% of patients had a family history of bronchial asthma and COPD.

### Analysis of influencing factors for pulmonary function grading in COPD high-risk groups

#### Univariate ordinal multi-classification logistic regression analysis

The results of the univariate analysis showed that cough and sputum production without getting a cold and family history had no statistically significant impact on pulmonary function grading and hence were not included in the subsequent multivariate analysis. In addition, we also provided the assignment tables for the corresponding reference groups. (Tables 3 and 4).

**Table 3:** Variable assignment table

Variable	Category	Assignment
Gender	Male	1
	Female	0
Age	21–49	1
	50–59	2
	60–69	3
	≥ 70	4
Consumption of cigarettes	0–14	1
	15–30	2
	≥ 30	3
BMI	< 18.5(very thin)	1
	18.5–23.9(normal)	2
	24-27.9(fattish)	3
	≥ 28(very fat)	4
Cough and produce phlegm without a cold	Yes	1
	No	0
Shortness of breath	No shortness of breath	1
	Shortness of breath when walking quickly on flat ground or climbing hills	2
	Shortness of breath when walking normally on flat ground	3
Use coal stove firewood	Yes	1
	No	0
Family history	Yes	1
	No	0

**Table 4:** Analysis of influencing factors for pulmonary function grading in COPD high-risk groups - Univariate ordinal multi-classification logistic regression analysis

Variables	Coefficient	OR value	95% confidence interval for the OR value	t value	P value
Gender male	0.74	2.095	(1.427,3.093)	3.751	< 0.001
Age 50–59 years	0.916	2.499	(1.469,4.304)	3.345	0.001
Age 60–69 years	1.359	3.894	(2.249,6.832)	4.804	< 0.001
Age ≥ 70 years	1.574	4.825	(2.81,8.404)	5.639	< 0.001
Consumption of cigarettes (packs per year) 15–30	-0.258	0.772	(0.476,1.25)	-1.049	0.294
Consumption of cigarettes (packs per year) ≥ 30	0.378	1.459	(0.988,2.158)	1.896	0.058
BMI 18.5–23.9	0.7	2.014	(1.292,3.165)	3.067	0.002
BMI 24.0–27.9	1.242	3.462	(2.05,5.893)	4.616	< 0.001
Have cough and sputum production without getting cold	0.632	1.881	(0.739,5.036)	1.306	0.191
Feeling shortness of breath when walking at a normal pace on level ground	-2.271	0.103	(0.067,0.156)	-10.602	< 0.001
Feeling shortness of breath when walking quickly on level ground or climbing gentle slopes	-4.05	0.017	(0.003,0.068)	-5.124	< 0.001
Use of firewood, grass, and coal stoves	-0.377	0.686	(0.484,0.972)	-2.119	0.034
Have family history of COPD	-0.076	0.927	(0.636,1.353)	-0.394	0.694

#### Multivariate analysis

The results of multivariate ordinal multi-classification logistic regression are shown in Table 5. Gender, age, shortness of breath, and the use of firewood, grass, and coal stoves were all influencing factors in pulmonary function grading. Among them, males were at greater risk of a higher grade of pulmonary function than females; those aged ≥ 60 years were at greater risk of a higher grade of pulmonary function than those aged 20–49 years; those “feeling short of breath when walking quickly on level ground or climbing gentle slopes” and those with “no shortness of breath” were at lower risk of a higher grade of pulmonary function than those “feeling short of breath when walking at a normal pace on level ground”, and those who used firewood, grass, and coal stoves were at lower risk of a higher grade of pulmonary function than those who did not.

**Table 5:** Analysis of influencing factors for pulmonary function grading in COPD high-risk groups - Multivariate ordinal multi-classification logistic regression analysis

Variables	Coefficient	OR value	95% confidence interval for the OR value	t value	P value
Gender male	0.561	1.753	(1.114,2.777)	2.412	0.016
Age 50–59 years	0.192	1.211	(0.636,2.324)	0.581	0.561
Age 60–69 years	0.795	2.215	(1.133,4.383)	2.308	0.021
Age ≥ 70 years	0.808	2.244	(1.113,4.573)	2.245	0.025
Consumption of cigarettes (packs per year) 15–30	-0.008	0.992	(0.577,1.702)	-0.029	0.976
Consumption of cigarettes (packs per year) ≥ 30	0.16	1.174	(0.739,1.866)	0.679	0.497
BMI 18.5–23.9	0.217	1.242	(0.731,2.116)	0.801	0.423
BMI 24.0–27.9	0.305	1.357	(0.734,2.515)	0.972	0.331
Feeling shortness of breath when walking at a normal pace on level ground	-2.414	0.089	(0.057,0.138)	-10.671	< 0.001
Feeling shortness of breath when walking quickly on level ground or climbing gentle slopes	-4.163	0.016	(0.002,0.066)	-5.061	< 0.001
Use of firewood, grass, and coal stoves	-0.579	0.561	(0.364,0.861)	-2.643	0.008

#### Parallelism test

The parallelism test is done to examine whether the effect of the independent variable values on the dependent variables is the same in each regression equation. The null hypothesis of the parallelism test is that the model conforms to parallelism. Therefore, a *P* value more than 0.05 indicates that we can accept the null hypothesis for the model, that is, it conforms to the parallelism test. Conversely, a *P* value less than 0.05 indicates that we can reject the null hypothesis for the model and that the model does not conform to the parallelism test. Parallelism is a prerequisite for ordinal logistic regression. The results of the parallelism tests in this study are shown in Table 6. All *P* values were greater than 0.05, except for the *P* values of BMI 3 and Shortness of breath 2, which were less than 0.05. It had little effect on the results and it can be considered that it conformed to the parallelism test.

**Table 6:** Parallelism test results

Variables	Gender male	Gender male	P value
Gender male	1.163	2	0.559
Age 50–59 years	3.375	2	0.185
Age 60–69 years	4.823	2	0.09
Age ≥ 70 years	0.861	2	0.65
Consumption of cigarettes (packs per year) 15–30	2.448	2	0.294
Consumption of cigarettes (packs per year) ≥ 30	2.545	2	0.28
BMI 18.5–23.9	0.964	2	0.618
BMI 24.0–27.9	10.125	2	0.006
Feeling shortness of breath when walking at a normal pace on level ground	13.236	2	0.001
Feeling shortness of breath when walking quickly on level ground or climbing gentle slopes	0	2	> 0.999
Use of firewood, grass, and coal stoves	5.583	2	0.061

## Discussion

The Chinese Adult Pulmonary Health Study (CPH) [8] estimated the incidence of COPD in adults aged 20 years and older in China as 8.6% and that of COPD in adults aged over 40 years as high as 13.7%, with a total of 99.9 million COPD patients in the country. The disease burden associated with COPD in China is substantial. [9] Although COPD is a respiratory disease with high morbidity, mortality, and disability, [10] the rates of early clinical diagnosis and early effective treatment are very low, delaying the optimal time for diagnosis and treatment. [11].

There is increasing interest in the origins of COPD because early prevention and early treatment can alter its clinical course. Martinez et al. [12] proposed an “operational definition” of early COPD based on intermediate endpoints that may eventually lead to pathological changes in the lungs. They defined the following conditions as necessary for early COPD: patients aged less than 50 years, who smoke ≥ 10 packs per year, and with any of the following abnormalities: (1) FEV1/FVC is less than the lower limit of normal; (2) abnormal CT imaging; or (3) decreased FEV1 (≥ 60 ml per year). Unfortunately, imaging data and continuous pulmonary function measurements are often not available for current patients or historical cohorts. GOLD 2020 recommends that people aged ≥ 40 years with any of these risk conditions are at high risk for COPD and should undergo further pulmonary function tests. [13] The importance of screening and early diagnosis of COPD cannot be overstated.

Primary-level screening and early diagnosis of COPD are the “first line” and “main battlefield” in China’s COPD prevention and treatment strategy, necessitating the concerted efforts of all medical personnel. [14] However, primary healthcare providers in the community often lack knowledge pertaining to the prevention and treatment of COPD, and the management of COPD patients is not standardized. [15, 16]

In this context, we established an early COPD screening system utilizing a two-way linkage between the community and the hospitals in Nanshan District in Shenzhen City. We conducted an extensive population survey and used a specifically developed questionnaire to identify individuals at high risk of COPD for further standard pulmonary function investigations. A total of 449 patients were diagnosed with COPD by pulmonary function tests. We calculated the prevalence of COPD in people aged 20 years and over in Nanshan District of Shenzhen City to be 8.98% using a base of 5000, which is similar to the COPD prevalence of 8.6% in Chinese adults aged 20 years and above, as obtained by CPH. [8].

As per classification of severity using GOLD criteria, we found 156 cases of GOLD I COPD, accounting for 34.74%; 169 cases of GOLD II COPD, accounting for 37.64%; 72 cases of GOLD III COPD, accounting for 16.04%; and 52 cases of GOLD IV COPD, accounting for 11.58%. Among them, 325 patients (72.38%) had GOLD I-II COPD, indicating that early COPD had a higher prevalence. The CPH survey [8] showed that the proportion of patients with GOLD I-II COPD was as high as 92.2% in China, and the vast majority of COPD patients had never received any treatment. Despite methodological differences, the results suggest that the prevalence of early COPD is on the rise rapidly and has reached a certain prevalence rate in the country’s general population.

It is worth noting that we also found that early COPD was more prevalent in people aged 50–59 years, those with severe respiratory symptoms, and those with a family history of bronchial asthma and COPD. Similar to this finding, another Chinese study has demonstrated that cigarette smoking, ambient air pollution, underweight, childhood chronic cough, parental history of respiratory diseases, and low education are major risk factors for COPD [17]. In the Copenhagen population cohort study, it was observed that more than half of the patients with early-stage COPD were active smokers who reported chronic respiratory symptoms more often and had significant impairment of pulmonary function. [18] In our study, multivariate ordinal multi-classification logistic regression of the diagnosed COPD cases showed that gender, age, shortness of breath, and use of firewood, grass, and coal stoves were all influencing factors for pulmonary function grading. Among them, males were at greater risk of a higher grade of pulmonary function than females; those aged ≥ 60 years were at greater risk of a higher grade of pulmonary function than those aged 20–49 years; those “feeling short of breath when walking quickly on level ground or climbing gentle slopes” and those with “no shortness of breath” were at lower risk of a higher grade of pulmonary function than those “feeling short of breath when walking at a normal pace on level ground”, and those who used firewood, grass, and coal stoves were at lower risk of a higher grade of pulmonary function than those who did not.

Unavoidably, this study has several limitations. First, there is a gender bias among the suspected high-risk individuals identified with questionnaires who had standard pulmonary function tests, as women were oversampled. Second, using only the FEV1/FVC ratio can lead to underdiagnosis of airway obstruction in persons aged 20–49 years and overdiagnosis in older adults. Third, we did not exclude patients with asthma from the study population, which may have led to an overestimation of the prevalence of COPD in younger populations. Finally, similar to other large-scale population surveys, our diagnosis of COPD was based solely on spirometry. Large-sample, multi-center research in the future can yield more convincing and significant findings for directing therapeutic practice.

## Conclusion

In conclusion, our data show that COPD is very common among adults in China. In particular, early COPD is prevalent among people aged 50–59 years, those with severe respiratory symptoms, and those with a family history of bronchial asthma and COPD. The use of screening questionnaires in combination with pulmonary function tests should be considered a COPD screening strategy that can be implemented at the primary level in China with the goal of preventing and early detecting the disease and reducing its incidence, disability, and mortality, thus making it a public health priority.

## Data Availability

All data generated or analysed during this study are included in this article. Further enquiries can be directed to the corresponding author.
